# HDAC1 and HDAC6 are essential for driving growth in IDH1 mutant glioma

**DOI:** 10.1038/s41598-023-33889-3

**Published:** 2023-08-01

**Authors:** Matthew C. Garrett, Rebecca Albano, Troy Carnwath, Lubayna Elahi, Catherine A. Behrmann, Merissa Pemberton, Daniel Woo, Eric O’Brien, Brett VanCauwenbergh, John Perentesis, Sanjit Shah, Matthew Hagan, Ady Kendler, Chuntao Zhao, Aditi Paranjpe, Krishna Roskin, Harley Kornblum, David R. Plas, Q. Richard Lu

**Affiliations:** 1 Department of Neurosurgery, University of Cincinnati College of Medicine, Cincinnati, OH 45267 USA; 2 University of Cincinnati College of Medicine, Cincinnati, OH 45267 USA; 3 Department of Molecular Cell and Developmental Biology, University of California Los Angeles, Los Angeles, CA 90095 USA; 4 Department of Cancer Biology, University of Cincinnati, Cincinnati, OH 45267 USA; 5 Department of Neurology, University of Cincinnati College of Medicine, Cincinnati, OH 45267 USA; 6 Division of Experimental Hematology and Cancer Biology, Department of Pediatrics, Cincinnati Children’s Hospital Medical Center, University of Cincinnati College of Medicine, Cincinnati, OH USA; 7 Department of Pathology and Laboratory Medicine, University of Cincinnati College of Medicine, Cincinnati, OH USA; 8 Bioinformatics Collaborative Services, Division of Biomedical Informatics, Cincinnati Children’s Hospital Medical Center, Cincinnati, OH USA

**Keywords:** Cancer, Cell biology, Drug discovery, Molecular biology, Oncology

## Abstract

Low-grade and secondary high-grade gliomas frequently contain mutations in the IDH1 or IDH2 metabolic enzymes that are hypothesized to drive tumorigenesis by inhibiting many of the chromatin-regulating enzymes that regulate DNA structure. Histone deacetylase inhibitors are promising anti-cancer agents and have already been used in clinical trials. However, a clear understanding of their mechanism or gene targets is lacking. In this study, the authors genetically dissect patient-derived IDH1 mutant cultures to determine which HDAC enzymes drive growth in IDH1 mutant gliomas. A panel of patient-derived gliomasphere cell lines (2 IDH1 mutant lines, 3 IDH1 wildtype lines) were subjected to a drug-screen of epigenetic modifying drugs from different epigenetic classes. The effect of LBH (panobinostat) on gene expression and chromatin structure was tested on patient-derived IDH1 mutant lines. The role of each of the highly expressed HDAC enzymes was molecularly dissected using lentiviral RNA interference knock-down vectors and a patient-derived IDH1 mutant in vitro model of glioblastoma (HK252). These results were then confirmed in an in vivo xenotransplant model (BT-142). The IDH1 mutation leads to gene down-regulation, DNA hypermethylation, increased DNA accessibility and H3K27 hypo-acetylation in two distinct IDH1 mutant over-expression models. The drug screen identified histone deacetylase inhibitors (HDACi) and panobinostat (LBH) more specifically as the most selective compounds to inhibit growth in IDH1 mutant glioma lines. Of the eleven annotated HDAC enzymes (HDAC1-11) only six are expressed in IDH1 mutant glioma tissue samples and patient-derived gliomasphere lines (HDAC1-4, HDAC6, and HDAC9). Lentiviral knock-down experiments revealed that HDAC1 and HDAC6 are the most consistently essential for growth both in vitro and in vivo and target very different gene modules. Knock-down of HDAC1 or HDAC6 in vivo led to a more circumscribed less invasive tumor. The gene dysregulation induced by the IDH1 mutation is wide-spread and only partially reversible by direct IDH1 inhibition. This study identifies HDAC1 and HDAC6 as important and drug-targetable enzymes that are necessary for growth and invasiveness in IDH1 mutant gliomas.

## Introduction

Sequencing studies from adult low-grade gliomas have revealed point mutations in the active sites of the IDH1 and IDH2 metabolic enzymes that bestow a novel enzymatic function of reducing alpha-ketoglutarate to the oncometabolite 2-hydroxyglutarate (2-HG)^1^. Many chromatin regulating enzymes (e.g. TET DNA demethylases and JMJ histone demethylases) require alpha-ketoglutarate as a co-factor and at high concentrations 2-HG can act as a competitive inhibitor leading to DNA and histone hypermethylation with resulting wide-spread gene down-regulation^2^. Since that discovery, the majority of research has focused on the effect of the IDH1 and IDH2 mutations on TET2 and DNA hypermethylation leaving histones relatively under-studied^3–5^. TET2 and IDH mutations are common and mutually exclusive in acute myelogenous leukemia^6^ (AML) and Tet2 and IDH1 knockout mice both spontaneously develop leukemia^7,8^. This suggests that in the case of AML, the IDH1 mutation is working primarily through inhibition of TET2 function. However, the tumorigenic mechanism of the IDH mutations in gliomas is less clear. TET2 mutations are rare in glioma and neither Tet2 nor IDH1 knockout mice spontaneously generate brain tumors.

Sequencing studies from histologically similar pediatric low-grade diffuse intrinsic pontine gliomas (DIPG) have revealed a single lysine to methionine substitution in the H3 histone subunit (H3K27M)^9–11^. In normal circumstances, the H3K27 lysine can either be acetylated (H3K27ac) or methylated (H3K27me3) with resulting opening or closing of the nucleosome respectively^11,12^. Mouse models show that the H3K27M mutation leads to increased histone acetylation and increased gene expression^9,10^. Interestingly, these studies also showed that the levels of H3K27 acetylation correlate with intracellular concentrations of alpha-ketoglutarate leading to the hypothesis that the IDH1/2 mutations, which cause alpha-ketoglutarate depletion, may lead to a corresponding H3K27 deacetylation^13^.

Following the discovery of the IDH1 mutation, it was hoped that inhibiting this mutant enzyme and decreasing the intra-cellular concentrations of 2-HG might lead to reversal of the epigenetic changes induced by the mutation and improved survival. Since then, several effective and highly specific IDH1/2 inhibitors have been developed^14^. Initial results from clinical trials in AML have been highly encouraging with both mouse models and clinical trials showing reversal of epigenetic changes and improved survival^15,16^. Unfortunately, data from gliomas has been more mixed. An initial study using an IDH1 mutant inhibitor in a xenotransplant model found increased survival and demethylation of the GFAP gene^17^. However, a follow-up study found no changes in growth or DNA/histone methylation after a prolonged treatment period^18^. Clinical trials in glioma have also been underwhelming with some reports of tumor stability but no reports of consistent tumor shrinkage^19^. In the case of glioma, it appears that additional therapies are needed to reverse the epigenetic dysregulation induced by the IDH1 mutant enzyme.

The histone deacetylase family is comprised of eleven genes (HDAC 1–11) and is responsible for removing acetyl groups from histone residues e.g. H3K27. Histone deacetylases (HDAC) are highly expressed in a wide-range of cancers and are highly drug-targetable making them a frequent topic for research studies^20–23^. Early clinical studies found that use of valproic acid (a well-known HDAC inhibitor and anti-seizure drug) was associated with improved survival in glioma patients^24^. Follow-up studies have been encouraging but mixed^25–30^. Interestingly, three recent studies found that HDAC inhibitors may be specifically effective in IDH1 mutant gliomas^31–33^. Histone deacetylases are ubiquitously expressed in various cell types and regulate multiple cellular processes. An effective cancer therapeutic would need to have some degree of specificity to slow tumor growth with tolerable side effects. In this study we investigate the effect of the IDH1 mutation on H3K27 acetylation and determine which HDAC genes are essential for growth in patient derived IDH1 mutant glioma cells.

## Results

### The IDH1 mutant enzyme leads to gene down-regulation, DNA hypermethylation, increased DNA accessibility and histone hypo-acetylation

Shortly after the discovery of the IDH1 mutation and the inhibitory effects of 2-HG on alpha-ketoglutarate dependent enzymes, it was widely hypothesized that the IDH1 mutation leads to tumorigenesis via chromatin dysregulation^2,34–36^. It was also thought that inhibition of the IDH1 mutation and 2-HG depletion might reverse this dysregulation. While clinical trials with IDH mutant inhibitors are ongoing, initial results are disappointing^19^. To address this shortcoming and find alternative therapeutic targets, we began with a multi-omics approach to characterize the multiple effects of the IDH mutation on chromatin structure using two previously published and validated models of IDH glioma: (1) IDH1 mutant over-expression on E12 murine embryonic neurospheres^37^. (2) An IDH1 murine knock-in glioma model (IDH1WT/NRAS/G12V-shp53,shATRX vs. IDH1mut-R132H, NRAS G12V-sh53, shATRX)^38^. Mouse and human models of IDH1 mutant glioma were validated by measuring 2-HG levels (Supplementary Fig. 1A).

**Figure 1:**
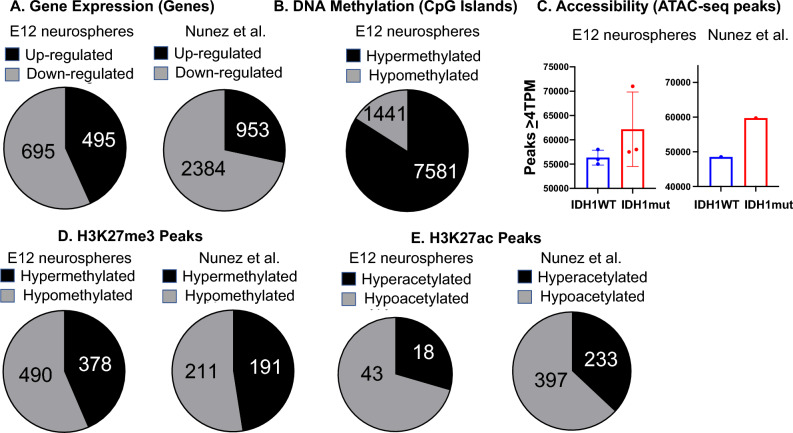
The IDH1 mutation leads to gene down-regulation, DNA methylation and histone hypo-acetyalation: three E12 murine neurosphere lines were created and infected with an IDH1 mutant over-expression vector (“E12 neurospheres”)^37^. Additionally cell lines were created from ex-planted mouse tumors from a previously published IDH1 mutant mouse glioma model (“Nunez et al.^38^”). (**A**) IDH1 wildtype and IDH1 mutant lines were compared using RNA-seq analysis revealing that IDH1 mutant samples are associated with gene down-regulation. (**B**) IDH1 wildtype and IDH1 mutant (N = 3 lines) murine E12 neurosphere lines underwent reduced bisulfite sequencing and the numbers of methylated islands were tallied confirming previous findings that IDH1 mutant samples are associated with hyper-methylation. (**C**) E12 neurospheres (N = 3) and the Nunez et al. glioma model (N = 1) underwent ATAC-seq analysis and revealed a non-significant trend for IDH1 mutant samples to have more peaks. (**D,E**) E12 neurospheres (N = 3) and Nunez et al. glioma cell lines (N = 3) underwent ChIP-seq with antibodies against H3K27me3 (**D**) and H3K27ac (**E**) revealing that the IDH1 mutation is associated with H3K27 hypoacetylation and a more modest trend towards H3K27 hypomethylation.

Given the role of the H3K27M mutation in diffuse intrinsic pontine glioma^13^ we elected to focus on the H3K27 histone residue. Consistent with other reports, over-expression of the IDH1 mutant enzyme led to gene down-regulation and DNA hyper-methylation in both mouse models (Fig. 1A,B). We also saw a non-significant increase in DNA accessibility using ATAC-seq in both models (Fig. 1C). Effects of the IDH1 mutant enzyme on H3K27 methylation showed equal amounts of hyper- and hypo-methylation (Fig. 1D). Regarding H3K27 acetylation, over-expression of the IDH1 mutant enzyme led to a decrease in histone acetylation (Fig. 1E). A panel of surgically resected samples (IDH1 wild-type glioma N = 4, IDH1 mutant glioma N = 4 and Control/Epilepsy tissue N = 4) were assayed with ChIP-seq for H3K27 modifications (H3K27me3 and H3K27ac). Unsupervised clustering of differential peaks revealed that H3K27ac peaks (but not H3K27me3 peaks) segregated IDH1 wildtype and IDH1 mutant samples into different groups, however we did not see a trend towards hypoacetylation in the IDH1 mutant samples (Supplementary Fig. 1B).

### Histone deacetylase inhibitors (HDACi) are selectively effective against endogenous IDH1 mutant lines

As seen in the previous results, the IDH mutation has multiple effects on different aspects of chromatin structure. To determine which of these effects are essential for growth and potentially drug-targetable, we undertook a drug screen of 106 chromatin modifying compounds. Previous studies indicate that endogenous serum-free glioma-sphere lines are the most accurate in vitro model^39^ and thus we used five patient-derived lines (IDH1 mutant-252^40^, BT142^41^, IDH1 wildtype-357, 385, 412^40^). Area under the curve (AUC) was calculated for each drug (Supplementary Fig. 2A,B). Drugs were ranked based on their selectivity for IDH1 mutant lines. Four of the top eight compounds that were most selective for IDH1 mutant cultures were histone deacetylase inhibitors (HDACi). Interestingly, triptolide, the second most selective compound, was independently verified by another group as effective against IDH1 mutant glioma cells^42^. Taken together these results indicate that the IDH1 mutant enzyme deacetylates histones and that the histone deacetylases may contribute essential and drug-targetable functions in IDH mutant glioma.

### Histone deacetylase inhibitors increase DNA accessibility and restore gene expression suppressed by mutant IDH1 while c227 (IDH1 mutant inhibitor) has minimal effect

**Figure 2:**
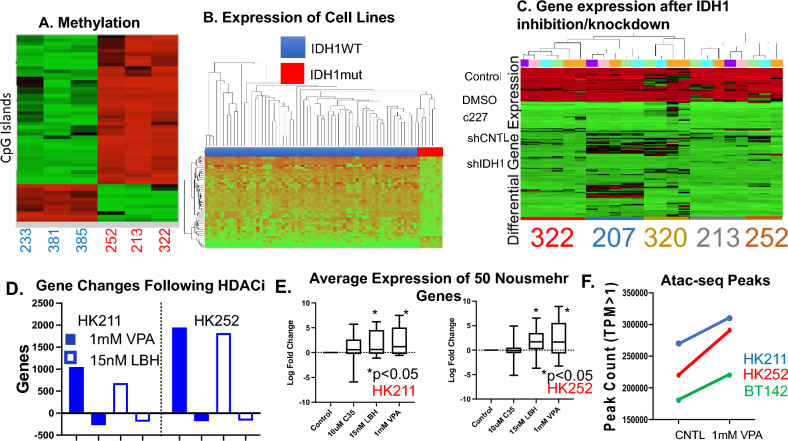
HDAC inhibitors lead to gene-up regulation in endogenous IDH1 mutant cell lines. (**A**) Three IDH1 wildtype (233, 381, 285) and three IDH1 mutant (252, 213, 322) lines underwent reduced bisulfite sequencing showing that consistent with the parent tumors, IDH1 mutant gliomaspheres are hypermethylated. (**B**) Previously published micro-array data^40^ from our collection of patient-derived lines showed that IDH1 mutant lines have a distinct expression profile of gene down-regulation. (**C**) Five IDH1 mutant lines (322, 207, 320, 213, and 252) underwent lentiviral knockdown and pharmacological inhibition of the IDH1 mutant enzyme. A heat-map was created of differentially expressed genes between IDH1 mutant and IDH1 wildtype samples. It showed neither pharmacological inhibition nor knockdown had any significant effect on gene expression. (**D**) In contrast, two well-known histone deacetylase inhibitors (valproic acid and panobinostat) lead to significant gene upregulation globally. (**E**) Significant gene up-regulation was also seen in a well-known previously published set of down-regulated methylated genes (e.g. Noushmehr genes). (**F**) Consistent with this gene up-regulation, ATAC-seq data shows that treatment with 1 mM valproic acid increased open DNA regions across multiple IDH1 mutant cell lines.

With the finding that histone deacetylase inhibitors may be promising therapeutics, we performed a multi-omics approach to determine the effect of HDAC inhibitors on the chromatin structure of IDH1 mutant gliomas. As an initial validation step, we confirmed that a collection of patient-derived IDH1 mutant lines recapitulated the gene down-regulation and DNA hypermethylation seen in their parent tumors (Fig. 2A,B). Consistent with other published reports^18^ pharmacological inhibition (c227) and genetic knock-down (shIDH1) of the IDH1 mutant protein had little effect on gene expression (Fig. 2C). In contrast, treatment with panobinostat (LBH) as well as valproic acid (a well-known and clinically used anti-epileptic and HDAC inhibitor) showed wide-spread gene up-regulation (Fig. 2D) and specifically showed significant up-regulation of published down-regulated and methylated genes (e.g. Noushmehr genes^43^) (Fig. 2E). There was also an observed increase in DNA accessibility based on ATAC-seq analysis (Fig. 2F). Differential analysis of CUT&RUN peaks showed that LBH treatment led to decreased numbers of H3K27me3 peaks and a smaller increase in H3K27ac peaks. Western blot analysis showed a dose dependent increase in total H3K27 acetylation indicating that there may be additional acetylation of existing H3K27 loci as opposed to the creation of new loci (Supplementary Fig. 1C–E). Taken together these results imply that histone deacetylase inhibitors are more effective than IDH mutant inhibition at reversing the genetic down-regulation that accompanies the IDH1 mutant gene.

### Histone deacetylase expression in IDH1 mutant gliomas

**Figure 3:**
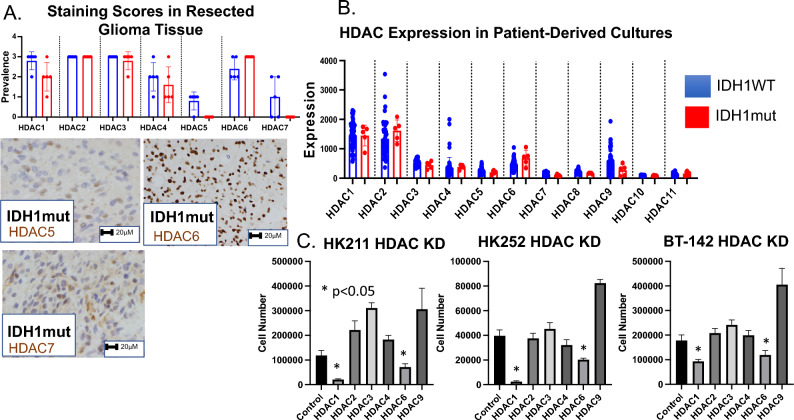
HDAC1 and HDAC6 are essential for growth in a panel of IDH1 mutant lines. (**A**) A panel of 10 surgical specimens (5 IDH1 wildtype and 5 IDH1 mutant) were stained with antibodies against HDAC proteins (HDAC1-7) and scored on a scale of 1–3 by a blinded neuropathologist. (**B**) A collection of 58 patient-derived cell lines (53 IDH1 wildtype, 5 IDH1 mutant) underwent expression analysis and the relative expression of each HDAC gene is shown. (**C**) Knock-down lentiviral ventors were designed against the 6 HDAC genes that were expressed in IDH1 mutant glioma tissue and cell lines (HDAC1-4, HDAC6, HDAC9) and tested on three IDH1 mutant lines. Relative growth was measured on day 14.

Clinical trials investigating the effects of non-specific pan-HDAC inhibitors on IDH WT gliomas have been limited by non-specific toxicity^20–22,44^. There are eleven documented histone deacetylases in the mammalian genome (HDAC1–11). By determining which of these HDAC genes are the most critical for growth it should be possible to identify more specific HDAC inhibitors that might be able to slow growth with more limited side effects**.** As an initial step, we determined which HDAC genes are highly expressed in IDH1 mutant gliomas. Ten glioma samples (5 IDH1 wildtype and 5 IDH1 mutant) were stained for various HDAC genes using internally validated antibodies (HDAC1, HDAC2, HDAC3, HDAC4, HDAC5, HDAC6 and HDAC7) and scored by a blinded neuropathologist (AK). Additional testing from multiple vendors was unable to find any specific staining with antibodies for HDAC8–11. This data was supplemented by RNA expression data from our collection of patient derived glioma lines. Staining of surgical samples and expression data showed high expression of HDAC1, HDAC2, HDAC3, HDAC4 and HDAC6 at the RNA and protein level (Fig. 3A,B). Short hairpin interference lentiviral vectors (shRNA) were obtained against the highly expressed HDAC genes and tested in three endogenous IDH1 mutant lines (211, 252, and BT-142). Cells were plated at equal density, infected with lentivirus, and then counted on day 14. Knock-down efficiency as well as the possibility of gene cross-regulation was tested with RNA-seq analysis and western blot (Supplementary Fig. 3A–D). Lentiviruses directed against HDAC1 and HDAC6 saw the greatest decrease in cell number (Fig. 3C). Two additional independent constructs against HDAC1 and HDAC6 were tested to rule out non-specific effects and confirmed decreased growth (Supplementary Fig. 3E).

### Gene targets of histone deacetylases and histone deacetylase inhibitors

**Figure 4:**
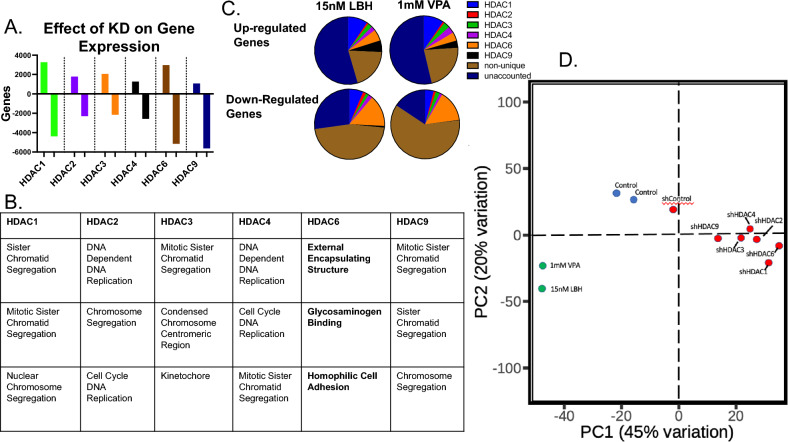
HDAC enzymes up-regulate DNA replication gene modules. HDAC6 targets a unique set of gene modules. (**A**) Each HDAC knock-down underwent RNA-seq analysis. The number of up-regulated and down-regulated genes are shown. (**B**) These genes underwent gene set enrichment analysis (Panther) the most enriched modules for each knockdown are shown. (**C**) A list of significantly changed (> 2 fold changed) genes from each of the drug treatment data sets was assigned to one of the following sub-groups: (1) unique to one of the HDAC knockdown sets, (2) “non-unique” i.e. shared by more than one HDAC knock-down gene set, or (3) “unaccounted” if the gene was not found in any of the HDAC knock-down sets). (**D**) RNA-seq datasets from each knockdown as well as drug treatment samples (1 mM VPA, and 15 nM LBH) underwent PCA analysis (HK252).

To dissect the genetic contribution of each HDAC gene, line HK252 was infected with each of the lentiviral HDAC knockdowns (HDAC1–4, HDAC6, and HDAC9) before undergoing RNA-seq analysis. Expression data from each knock-down was compared to an empty vector. Differentially expressed genes underwent gene set enrichment analysis. The number of up-regulated and down-regulated genes are plotted (Fig. 4A, Supplementary Fig. 3F,G). Despite the previous finding that pan-histone deacetylase inhibitors lead to wide-spread gene up-regulation (Fig. 2D), data from the individual knockdowns indicate that the HDAC genes have both activating and repressive functions (Fig. 4A). Gene set enrichment analysis (GSEA) revealed a large set of gene modules that were positively regulated by the HDAC genes (Fig. 4B). Most of these enriched gene sets were heavily over-lapping and involved DNA replication and chromosome division except for HDAC6 which involved gene sets involved with extracellular matrix and adhesion (Fig. 4B). To reconcile the different expression results seen in the drug-treated (LBH and VPA) and the HDAC knockdown samples, HK 252 was again treated with 1 mM VPA for 5 days or 15 nM LBH for 3 days before undergoing RNA-seq analysis. The resulting datasets underwent PCA analysis showing that the HDAC knockdowns and the drug-treated samples clustered separately (Fig. 4D). Using bio-informatic methods to dissect the gene changes seen following drug treatment, each significant gene change (twofold change) seen after drug treatment was assigned into one of the following groups: unique to one HDAC gene, “non-unique” (i.e. common to more than one HDAC gene), or “unaccounted” (i.e. gene was not seen in any of the HDAC knockdowns). Following this analysis, it was noted that the majority of the down-regulated genes seen in the drug-treated samples could be accounted for with one or several of the HDAC genes with HDAC6 being the greatest contributor. In contrast, most of the up-regulated genes in the drug-treated samples could not be accounted for with the available HDAC knockdown data suggesting that more complex interactions may be involved (Fig. 4C).

### HDAC1 and HDAC6 lead to more infiltrative and diffuse tumor growth

**Figure 5:**
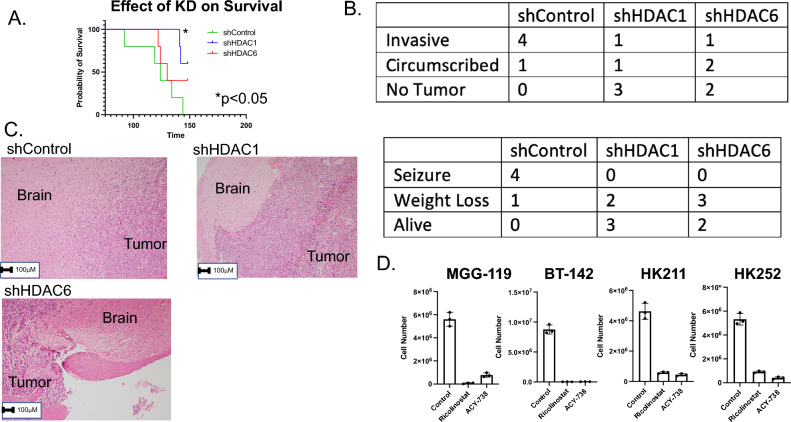
HDAC1 and HDAC6 drive invasiveness in an in vivo model of IDH1 mutant glioma. (**A**) 10,000 BT-142 cells infected with the following lentiviral constructs (shControl, shHDAC1, and shHDAC6) were implanted in the caudate of NSG mice and allowed to grow until symptoms developed (N = 5 per cohort). (**B**) Neurological symptoms were recorded (e.g. “Weight loss,” “Seizures”) and following development of symptoms, mice were euthanatized. Following euthanasia, the brains were removed, sectioned, stained with hematoxylin and eosin, and graded by a blinded neuropathologist (MH) for “Invasive,” “Non-Invasive,” or “No tumor seen”. (**C**) Representative sections are shown. (**D**) Given the important role of HDAC6 in IDH1 mutant gliomas, two HDAC6 inhibitors (10 μM Ricolinostat, 10 μM ACY-738) were tested on a panel of four IDH1 mutant glioma lines for 7 days to determine the effect on cell number.

Following up on the in vitro growth data, BT-142 cells were infected with one of three lentiviral constructs (empty vector/shControl, shHDAC1, shHDAC6) before being injected into the striatum of nod-SCID-gamma null mice (10,000 cells/mouse, N = 5 mice per cohort). Mice were euthanized upon development of neurological symptoms. shHDAC1 mice showed a significant survival advantage over shControl. shHDAC6 showed a trend towards improved survival that did not reach statistical significance (Fig. 5A). Interestingly the phenotypic observations indicated that shControl mice tended to develop seizure symptoms, while shHDAC1 and shHDAC6 developed weakness and weight-loss symptoms (Fig. 5B). Following euthanasia, the brains were extracted, sectioned, and stained with hematoxylin and eosin. The slides were then scored by a blinded neuropathologist (MH) who determined that shControl tumors were more invasive than shHDAC1 and shHDAC6 tumors (Fig. 5B,C.). The previous findings identify HDAC6 as a potential specific target for IDH1 mutant gliomas. To test this hypothesis, two reported specific HDAC6 inhibitors (10 μM Ricolinostat, 10 μM ACY-738) were tested on a panel of IDH1 mutant glioma lines and showed high efficacy against these lines (Fig. 5D).

## Discussion

Despite being only a single amino acid substitution, the R132H IDH1 mutation has multiple far-reaching effects on the immunogenicity, metabolic and epigenetic state of the cell. Given these alterations to cellular behavior it is tempting to design specific therapy that might exploit these cellular effects. Studies indicate that the IDH1 mutation may be the initial mutation that initiates tumorigenesis and thus resides in every tumor cell^45^. This makes it a promising vaccine target and to that end vaccine clinical trials are ongoing^46,47^. Working against this strategy is evidence that the IDH1 mutation and 2-HG are immune suppressive and may blunt an immune response^48,49^. Despite being an oncogene, the IDH1 mutant enzyme causes significant metabolic strain on numerous cellular pathways. Subsequently over-expression^50^ and mouse knock-in studies^51^ have found that the IDH1 mutation leads to slower growth and cell death. This presents an opportunity to exploit these vulnerabilities. Seltzer et al.^52^ found that the IDH1 mutant enzyme depletes cellular stores of glutamate and makes cells vulnerable to glutaminase inhibitors. Tateishi et al.^18^ found that the IDH1 mutant enzyme depletes the cell of NAD+ making cells vulnerable to NAMPT inhibition. The efficacy of radiation on IDH1 mutant tumors is controversial^53^ and in vitro studies on the effect of the IDH1 mutation on DNA repair pathways and radiation sensitivity have been mixed^3,37,38,54^. While the tumorigenic mechanism of the IDH1 mutation has never been definitively proven, the most accepted hypothesis is that 2-HG-based inhibition of chromatin-modifying enzymes leads to pathologic gene expression^55,56^. While there was initial hope that inhibition of the IDH1 mutant enzyme might be able to reverse these changes, this study and others have found the effects on gene expression and growth underwhelming^19^. The purpose of the current study is to find additional compounds that could potentially reverse some of the pathologic epigenetic changes induced by the IDH1 mutation. To that end, an unbiased drug screen identified LBH/Panobinostat, a pan-HDAC inhibitor, as the most selectively effective compound against IDH1 mutant glioma lines. Follow-up experiments revealed that LBH up-regulates many of the repressed genes and increases chromatin accessibility.

The DNA methylation status of the MGMT promoter in gliomas was the first clinically used epigenetic biomarker and since that time much of the focus on glioma epigenetics has been on DNA methylation. However, the discovery of the H3K27M mutation in diffuse intrinsic pontine gliomas has highlighted the importance of histone modification and the H3K27 locus specifically in gliomagenesis. While the exact mechanism of the H3K27M mutation is not known, mouse knock-in models show that the H3K27M mutation is associated with histone hyper-acetylation and is interestingly incompatible with the R132H IDH1 mutation^13^. Chung et al. also reported that levels of H3K27 acetylation were correlated with levels of alpha-ketoglutarate. The IDH1 mutant enzyme is known to deplete cellular levels of alpha-ketoglutarate^37^ and thus it would be predicted this would lead to H3K27 hypo-acetylation. In the current study we used two IDH1 over-expression models to determine the effect of the IDH1 mutant enzyme on chromatin. Similar to previous reports we found that the IDH1 mutation was associated with gene down-regulation, DNA hypermethylation^43^ and a non-significant trend towards increased DNA accessibility^57^. Consistent with our prediction, the IDH1 mutation was associated with H3K27 hypoacetylation. Contrary to predictions from other in vitro over-expression studies we did not observe increased H3K27 hyper-methylation (Fig. 1D). There are several explanations for this. One explanation is that previous studies used western blots which measures the total amount of H3K27me3 molecules while in this study we used ChIP-seq which measures the number of H3K27me3 loci. It might be that the IDH1 mutation leads to more deposition of H3K27me3 without leading to any additional methylated loci. A similar phenomenon was observed in DIPG where H3K27M mutation led to histone hyper-acetylation but no new enhancer sites ^11^.

In general, this study finds that the magnitude of the effects on H3K27 modification are noticeably smaller than the effects on DNA methylation. However, the relative size of the effect may conceal its biological importance. DNA methylation changes are thought to be slow requiring multiple cell divisions^34^. Studies have investigated the possibility of restoring the methylation state of IDH1 gliomas by inhibiting the DNMT family of DNA methylases with decitabine^58^. While eventually effective, the therapy is slow with non-specific off-target toxicity. In contrast, targeting histone architecture has the potential to yield faster efficacy with less toxicity. Consistent with this idea, sub-group analysis from the Stupp^59^ trial found that valproic acid use (a known HDAC inhibitor) was associated with improved survival^24^. Follow-up studies investigating the survival benefits of valproic acid in glioma have been mixed but generally encouraging^25–30^. It should be noted that these studies used mixed populations of IDH1 wildtype and IDH1 mutant gliomas with IDH1 wildtype likely being the majority. Recently three studies have confirmed our findings that HDAC inhibitors have specific efficacy against IDH1 mutant gliomas^31–33^ providing a rationale for a follow-up clinical study restricted to the IDH1 mutant sub-group. Interestingly, phase I and II clinical trials with newer non-selective pan-HDAC inhibitors (e.g. vorinostat^22,60^, panobinostat^44^ and romidepsin^61^) have been less encouraging with no demonstrated efficacy and dose-limiting side effects. Histone deacetylase activity is likely essential for cellular function and non-selective inhibition of all histone deacetylases will likely be associated with unwanted toxicity. To address this limitation, this study identifies the specific members of the HDAC family that are essential for IDH1 mutant glioma growth (i.e. HDAC1 and HDAC6). While many of the HDAC target genes and gene modules were heavily over-lapping, HDAC6 seemed to target more unique genes and gene modules. The unique structure and gene targets of the HDAC6 enzyme lends itself to specific inhibitors and several specific HDAC6 inhibitors have been developed as anti-cancer agents in clinical trials (clinicaltrials.gov NCT03008018, NCT02935790, NCT01323751).

This study has several limitations and unexpected findings that should be mentioned. First, BT-142 is one of the few IDH1 mutant cell lines that transplants into immune-deficient mice. However, the cell line is hemizygous for the IDH1 mutation and thus produces lower levels of 2-HG. Nevertheless, its gene expression pattern still clusters with other IDH1 mutant lines. Second, despite numerous attempts it appears that the IDH1 mutant lines used in this study are resistant to CRISPR manipulations, making genetic rescue experiments challenging. Interestingly, while over-expressing the IDH1 mutant enzyme did seem to decrease the number H3K27 acetylated loci, there was a fair amount of variability between samples indicating that this hypoacetylation may be affected by additional unidentified variables. Supporting that possibility, we did not see a consistent histone acetylation signature among a panel of IDH1 wildtype, IDH1 mutant and epilepsy surgical samples. In contrast the signal for DNA methylation is much stronger and surgical samples seem to have a more consistent DNA methylation signature^43^. Other studies have identified transcriptional repressor activity with histone deacetylases, however this study found that in an IDH1 mutant glioma model (HK252), the HDAC genes activate a set of chromosome organization and DNA replication gene modules. Interestingly, expression data from histone deacetylase inhibitors did reveal a set of up-regulated genes that were not seen in the HDAC knockdowns. One possible explanation for this observation is that because HDAC inhibitor compounds inhibit multiple HDAC proteins, there may be combinatorial effects that are not seen in single gene knock-down experiments. Consistent with this possibility is the observation that many of the HDAC enzymes are redundant and are therefore able to compensate for each other^62^. Another possibility is that HDAC enzymes have multiple biological functions in addition to histone deacetylation (e.g. protein modification). Gene knock-down and enzyme inhibition would likely affect these mechanisms differently.

In conclusion, histone deacetylase inhibitors offer a promising therapy for multiple cancers. Recent drug screens have shown that IDH1 mutant gliomas may be especially sensitive to these compounds. The key to success will be to tailor specific treatment to slow tumor growth while avoiding dose-limiting toxicities.

## Methods

### In vitro drug screen

A drug screen of 106 known epigenetic compounds targeting 35 genes and 19 epigenetic mechanisms (e.g. DNA methylation, DNA demethylation, histone methylation, histone demethylation, histone acetylation and histone deacetylation) was conducted against two IDH1 mutant (252 and BT-142) and three IDH1 wildtype lines (357, 385, 412) at four drug concentrations (10 nM, 100 nM, 1 μM, 10 μM) for 3 days. Viable cell number was estimated by MTT. Average IDH1mut viability was compared to average IDH1WT viability and displayed as area under the curve (IDH1WT/IDH1mut). Drugs were then ranked by compound.

### Generation of E12 neural progenitor cell lines

Timed pregnant dams (C56Bl) were sacrificed on E12. The embryos were removed from the uterus and the cortices were dissected free. Three distinct lines were created each by mincing three cortices per line and placing them in serum-free neurosphere media^63^ until neurospheres appeared. These three neural progenitor lines were then infected with either an IDH1 mutant lentivirus^37^ or a pUltra control. 2-HG levels for these lines as well as other IDH1 mutant lines were confirmed by LC–MS as previously described^37^. These lines were then maintained in culture for twelve passages before being processed for RNA-seq, ATAC-seq, methyl-seq and Chip-seq (H3K27ac, H3K72me and IgG).

### HDAC lentiviral knock-down experiments and drug treatment

Lentiviral plasmids with hairpin RNA sequences against HDAC genes were obtained from Sigma and were used to create lentiviral particles (HDAC1:CGGTTAGGTTGCTTCAATCTA/TATCCCGTAGGTCCCCAGT, HDAC2:CAGTCTCACCAATTTCAGAAA, HDAC3:CAAGAGTCTTAATGCCTTCAA, HDAC4:GCCAAAGATGACTTCCCTCTT HDAC6:CGGTAATGGAACTCAGCACAT/ AACCGCAAGCTGCATCCTG, HDAC9: CAAACTGCTTTCGAAATCTAT). 200,000 cells were incubated in 2 ml of serum-free media with 12ul of lentiviral particles. After 24 h the supernatant was removed and replaced with neurosphere media. 48 h later the media was replaced with fresh neurosphere media supplemented with 0.5ug/ml of puromycin. Knock-down was confirmed based on RNA-seq and western blots (Supplementary Fig. 3A,B). Western blots were cropped for clarity. Full-length plots are shown to demonstrate antibody specificity (Supplementary Fig. 3C,D). Based on preliminary in vitro experiments, drug doses of 15 nM LBH and 1 mM VPA were chosen to achieve a cell viability of ~ 60%. Cells underwent 3 days of drug treatment unless otherwise stated. Cells were treated with the HDAC6 inhibitors, 10 μm Ricolinostat or 10 μM ACY-738 for 7 days. All HDAC antibodies were obtained from Cell Signaling Technologies (HDAC1 10E2, HDAC2, 3F3, HDAC3 7G6C5, HDAC4 D15C3, HDAC6 D2B10).

#### Generation of libraries

All human samples were provided by the University of Cincinnati Biorepository in a de-identified manner and were designated “Not human research” by the local IRB. All methods were carried out in accordance with relevant guidelines and regulations. All data is available at: https://github.com/TroyCarnwath/garrett_lab.

#### ATAC-Seq

ATAC-seq libraries were created as described previously^64^. Briefly, 50,000 cells were spun down at 500×*g* for 5 min at 4 °C. Cells were resuspended in ATAC-resuspension buffer containing 0.1% NP40, 0.1% tween-20, and 0.01% digitonin and incubated on ice for 3 min. Cells were washed with ATAC-resuspension buffer containing 0.1% tween-20. Nuclei were pelleted at 500×*g* at 4 °C for 10 min. Nuclei were resuspended in transposition reaction mix containing TD 2× reaction buffer (Illumina, #20034197), TDE1 Nextera Tn5 Transposase (Illumina, #20034197), and nuclease free water. The reaction was incubated at 37 °C in a water bath for 30 min. Immediately following transposition, DNA was purified with a Qiagen MinElute PCR Purification Kit (Qiagen, #28004). Following purification, transposed DNA was mixed with NEBNext high-Fidelity 2× PCR Master Mix (NEB, #M0541S), AD1_noMX and AD2.1–2.16 barcoded primers, and nuclease free water. Samples were amplified for 12 cycles. Immediately following amplification, DNA was purified using a Qiagen MinElute PCR Purification Kit. ATAC-seq reads in FASTQ format were first subjected to quality control to assess the need for trimming of adapter sequences or bad quality segments. The programs used in these steps were FastQC v0.11.7, Trim Galore! v0.4.2 and cutadapt v1.9.1. The trimmed reads were aligned to the reference human genome version GRCh38/hg38 with the program HISAT2 v2.0.5. Aligned reads were stripped of duplicate reads with the program sambamba v0.6.8. Peaks were called using the program MACS v2.1.2 using the broad peaks mode. To obtain the consensus set of unique peaks, called peaks from all samples are merged at 50% overlap using BEDtools v2.27.0. The consensus peaks, originally in BED format were converted to a Gene Transfer Format (GTF) to enable fast counting of reads under the peaks with the program featureCounts v1.6.2. Each feature in the GTF file has the value "peak" on the second column. Peaks located on chromosomes X, Y and mitochondrial DNA are excluded from further analysis. Raw read counts are normalized with respect to library size and transformed to log2 scale using rlog() function in R package DESeq2 v1.26.0.

#### RNA-Seq

RNA (> 200 nucleotides) was purified from cells using the Quick-RNA MiniPrep Plus Kit (Zymo Research, #R1057). The quality control check on RNA-seq reads was performed with FastQC v0.11.7. Adapter sequences and bad quality segments were trimmed using Trim Galore! v0.4.2 and cutadapt v1.9.1. The trimmed reads were aligned to the reference human genome version GRCh38/hg38 with the program STAR v2.6.1e. Duplicate aligned reads were removed using the program sambamba v0.6.8. Gene-level expression was assessed by counting features for each gene, as defined in the NCBI's RefSeq database. Read counting was done with the program featureCounts v1.6.2 from the Rsubread package. Raw counts were normalized with respect to library size and transformed to log2 scale using rlog() function in R package DESeq2 v1.26.0.

#### Cut and Run/ChIP-seq

CUT&RUN was carried out using the CUT&RUN Assay Kit (cell signaling technology, #86652). Briefly, cells or tumor tissues were dissociated into single-cells and bound to Concanavalin A-coated magnetic beads, permeabilized with digitonin Buffer, and then incubated with primary antibody on a rotator overnight at 4 °C. The antibodies used were: H3K27ac (cell signaling technology, #8173), H3K27me3 (cell signaling technology, # 9733), IgG control (cell signaling technology, #3900). Cell-bead slurry was washed twice with Digitonin Wash, incubated with Protein A/G-MNase (pAG-MN) for 1 h at 4 °C, and then washed twice with Dig Wash. Slurry was then placed on a 4C block and MNase digestion was activated by CaCl2. After 30-min, the reaction was stopped with EGTA-STOP Buffer and fragments were released by incubation at 37 °C for 10 min. DNA was recovered from the supernatant after a 5-min centrifugation at 16,000*g* and purified via Phenol–Chloroform–isoamyl alcohol (Sigma, #145 p3803) extraction and ethanol precipitation essentially as described^65,66^. The resulting DNA was constructed into a library using NEBNext Ultra DNA Library Prep Kit (New England Biolabs, #E7645) following the manufacturer’s instructions. The libraries were sequenced in a 100-base pair pair-end run on the Next Seq 2000 (Illumina). The raw and processed data are provided at the Gene Expression Omnibus database (GEO, http://www.ncbi.nlm.nih.gov/geo). Read quality was assessed using FastQC v0.11.7. Adapter sequences and bad quality segments were removed using Trim Galore! v0.4.2 and cutadapt v1.9.1. The trimmed reads were aligned to the reference human genome version hg38 using Bowtie2 v2.3.4.1 with parameters "--dovetail --local --very-sensitive-local -I 10 -X 700 -p 12 --no-unal --no-discordant --no-mixed". Aligned reads in IgG samples were stripped of duplicate reads with the program sambamba v0.6.8. Peaks were called with MACS v2.1.4 using the parameters “-g hs -q 0.1” for H3K27ac samples and “--broad -g hs -q 0.1” for H3K27me3 samples. BEDtools v2.27.0 were used to obtain consensus peaks in two steps. In the first step, common peaks among samples from same group were obtained by selecting peaks which are detected in at least 75% of samples from same group. Common peaks in each group from first step were merged at 50% overlap to obtain unique consensus peaks from all groups. Consensus peaks, originally in BED format were converted to a Gene Transfer Format (GTF) and used for read quantification under the peaks with featureCounts v1.6.2 from Rsubread package. Peaks located on chromosomes X, Y and mitochondrial DNA are excluded from further analysis. Differential analysis between groups of samples were assessed with the R package DESeq2 v1.26.0. Plots were generated using the ggplot2 package and base graphics in R.

#### Xenotransplant model

Eight-week-old Nod-SICD-gamma null (NSG) mice were placed under isoflurane. The scalp was incised and 10,000 cells/3 μl of BT-142 cells (shControl, shHDAC1, shHDAC6) were injected into the striatum. Mice were monitored for the development of tumor symptoms. All methods were carried out in accordance with an approved protocol from the local IACUC of the University of Cincinnati with relevant guidelines and regulations. All surviving mice were sacrificed after 21 weeks. Following sacrifice, mice underwent transcardiac perfusion and the brains were removed and placed in 10% formalin for 3–7 days before being moved to a 30% sucrose solution. Brains were then sectioned and stained with hemotoxylin and eosin.

### Sequencing data

The datasets generated and/or analyzed during the current study are available in the Garrett Lab Data Directory (https://github.com/TroyCarnwath/garrett_lab). All experimental protocols were approved by the University of Cincinnati IRB committee. This study used de-identified patient specimens and was designated “Not human research” 19-02-25-02 (5/3/2019). Consent is not applicable.

### Human samples

All human samples are provided by the University of Cincinnati Biorepository which serves to provide de-identified patient specimens of the desired tissue type with clinical and demographic information. This project was submitted to the IRB of the University of Cincinnati and was designated “Not human research” (2019-0155). All methods were carried out in accordance with relevant guidelines and regulations.

### Animal research

All protocols involving xenotransplants in mice, including mouse housing and management were reviewed and approved by the local IACUC of University of Cincinnati (05-04-04-01 issued 1-11-2019 and 20-07-16-02 issued 08-28-2020). All methods were carried out in accordance with relevant guidelines and regulations. We have reviewed the ARRIVE guidelines including study design, sample size, inclusion/exclusion criteria, randomization, blinding, outcome measures, statistical methods, experimental animals, experimental procedures, and results and are consistent with these guidelines.

## Supplementary Information

Supplementary Figure 1.

Supplementary Figure 2.

Supplementary Figure 3.

## Data Availability

The datasets generated and/or analyzed during the current study are available in the Garrett Lab Data Directory (https://github.com/TroyCarnwath/garrett_lab).
