# Supplementary material for: HDAC1 and HDAC6 are essential for driving growth in IDH1 mutant glioma
Source: Sci Rep. 2023 Aug 1;13:12433. doi: 10.1038/s41598-023-33889-3 (PMC10394035; doi:10.1038/s41598-023-33889-3)
Supplement: Supplementary file 1 — Supplementary Figure 1. [file 41598_2023_33889_MOESM1_ESM.pptx]

## Slide 1
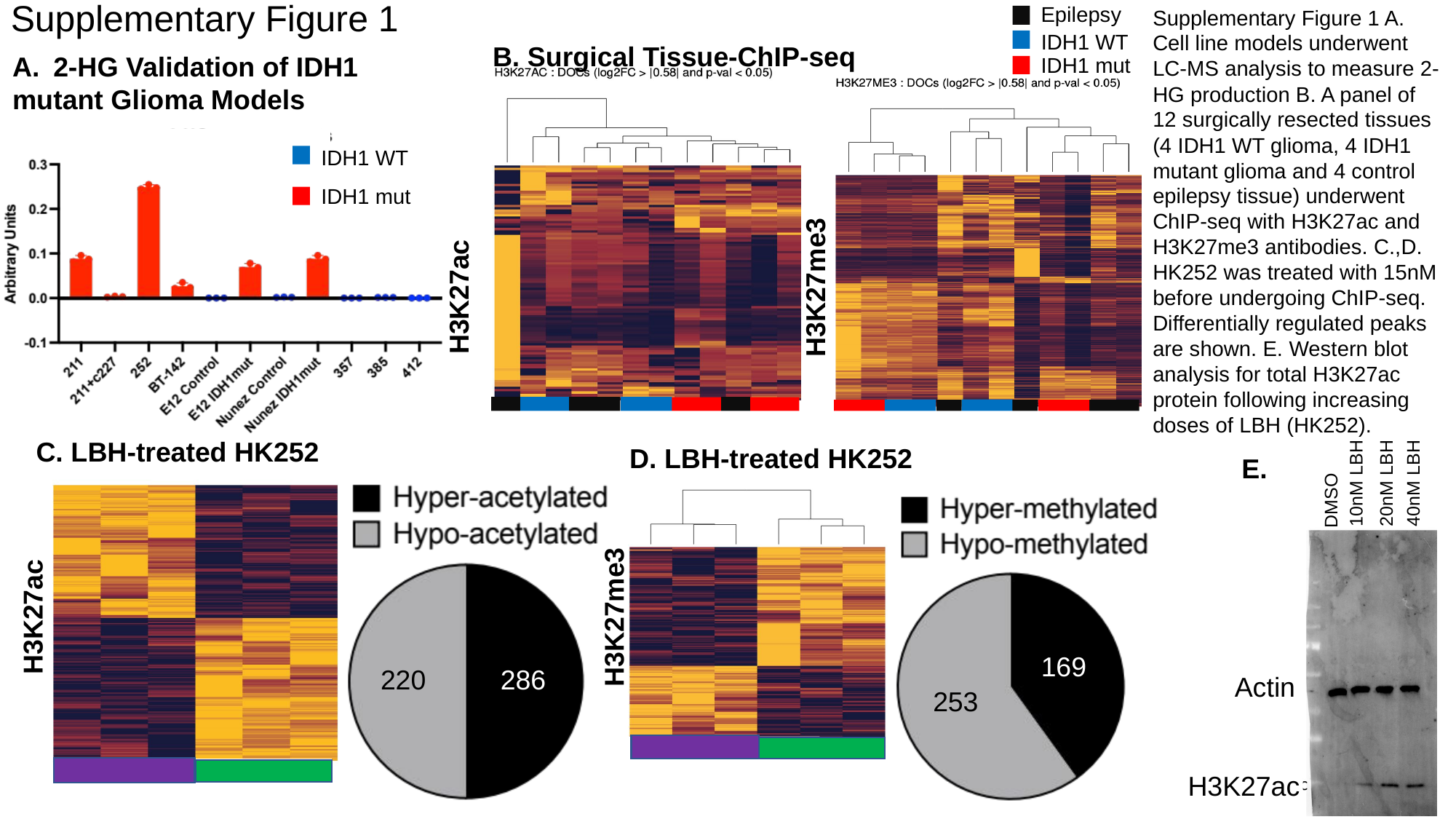

Supplementary Figure 1
Epilepsy
Supplementary Figure 1 A. Cell line models underwent LC-MS analysis to measure 2-HG production B. A panel of 12 surgically resected tissues (4 IDH1 WT glioma, 4 IDH1 mutant glioma and 4 control epilepsy tissue) underwent ChIP-seq with H3K27ac and H3K27me3 antibodies. C.,D. HK252 was treated with 15nM before undergoing ChIP-seq. Differentially regulated peaks are shown. E. Western blot analysis for total H3K27ac protein following increasing doses of LBH (HK252).
IDH1 WT
B. Surgical Tissue-ChIP-seq
2-HG Validation of IDH1
mutant Glioma Models
IDH1 mut
IDH1 WT
IDH1 mut
H3K27me3
H3K27ac
C. LBH-treated HK252
D. LBH-treated HK252
E.
10nM LBH
20nM LBH
40nM LBH
DMSO
H3K27ac
H3K27me3
169
220
286
Actin
253
H3K27ac
