# Supplementary material for: HDAC1 and HDAC6 are essential for driving growth in IDH1 mutant glioma
Source: Sci Rep. 2023 Aug 1;13:12433. doi: 10.1038/s41598-023-33889-3 (PMC10394035; doi:10.1038/s41598-023-33889-3)
Supplement: Supplementary file 2 — Supplementary Figure 2. [file 41598_2023_33889_MOESM2_ESM.pptx]

## Slide 1
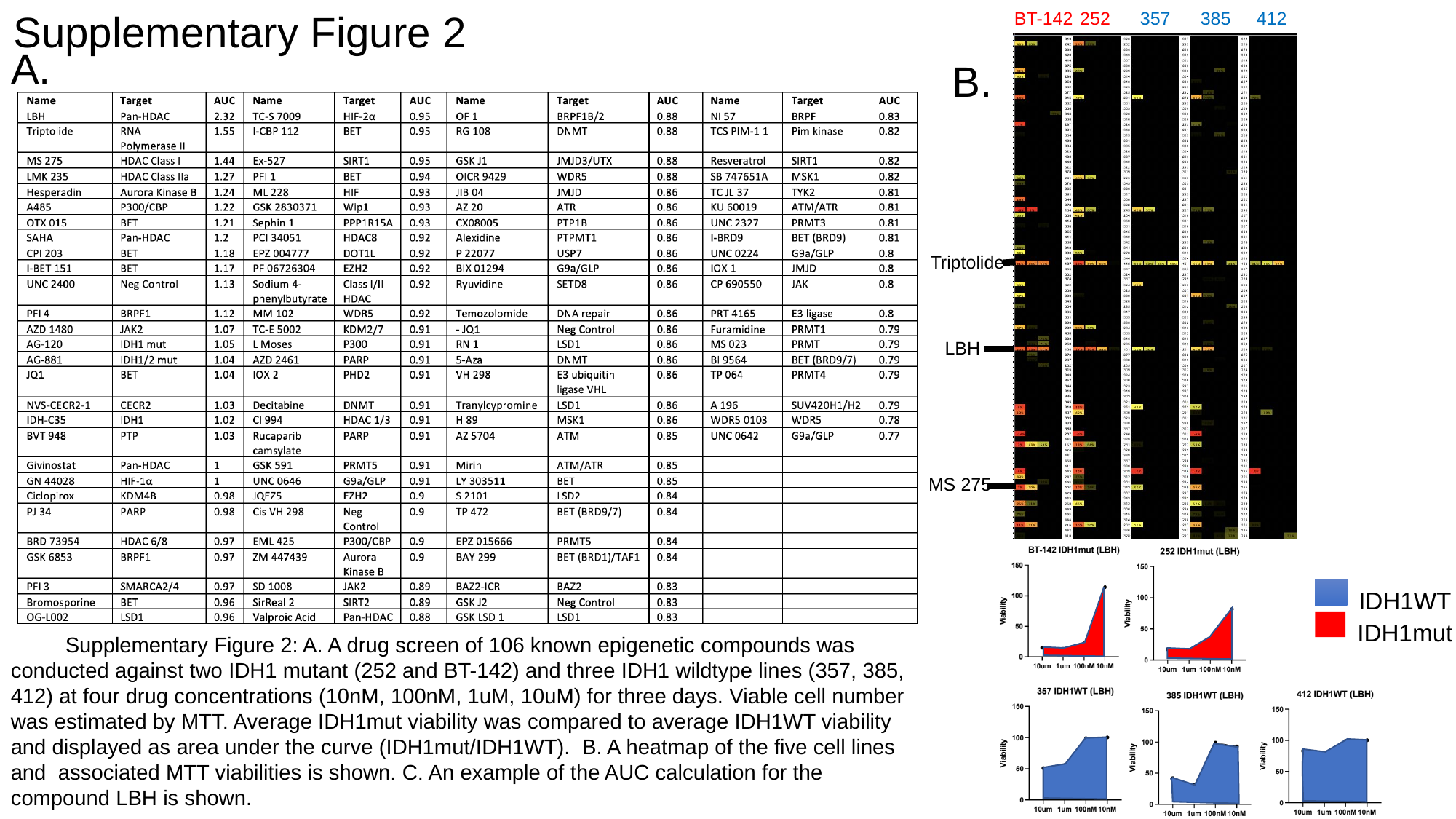

Supplementary Figure 2
BT-142
252
357
385
412
A.
B.
Triptolide
LBH
MS 275
IDH1WT
IDH1mut
Supplementary Figure 2: A. A drug screen of 106 known epigenetic compounds was conducted against two IDH1 mutant (252 and BT-142) and three IDH1 wildtype lines (357, 385, 412) at four drug concentrations (10nM, 100nM, 1uM, 10uM) for three days. Viable cell number was estimated by MTT. Average IDH1mut viability was compared to average IDH1WT viability and displayed as area under the curve (IDH1mut/IDH1WT). B. A heatmap of the five cell lines and associated MTT viabilities is shown. C. An example of the AUC calculation for the compound LBH is shown.
