# Supplementary material for: HDAC1 and HDAC6 are essential for driving growth in IDH1 mutant glioma
Source: Sci Rep. 2023 Aug 1;13:12433. doi: 10.1038/s41598-023-33889-3 (PMC10394035; doi:10.1038/s41598-023-33889-3)
Supplement: Supplementary file 3 — Supplementary Figure 3. [file 41598_2023_33889_MOESM3_ESM.pptx]

## Slide 1
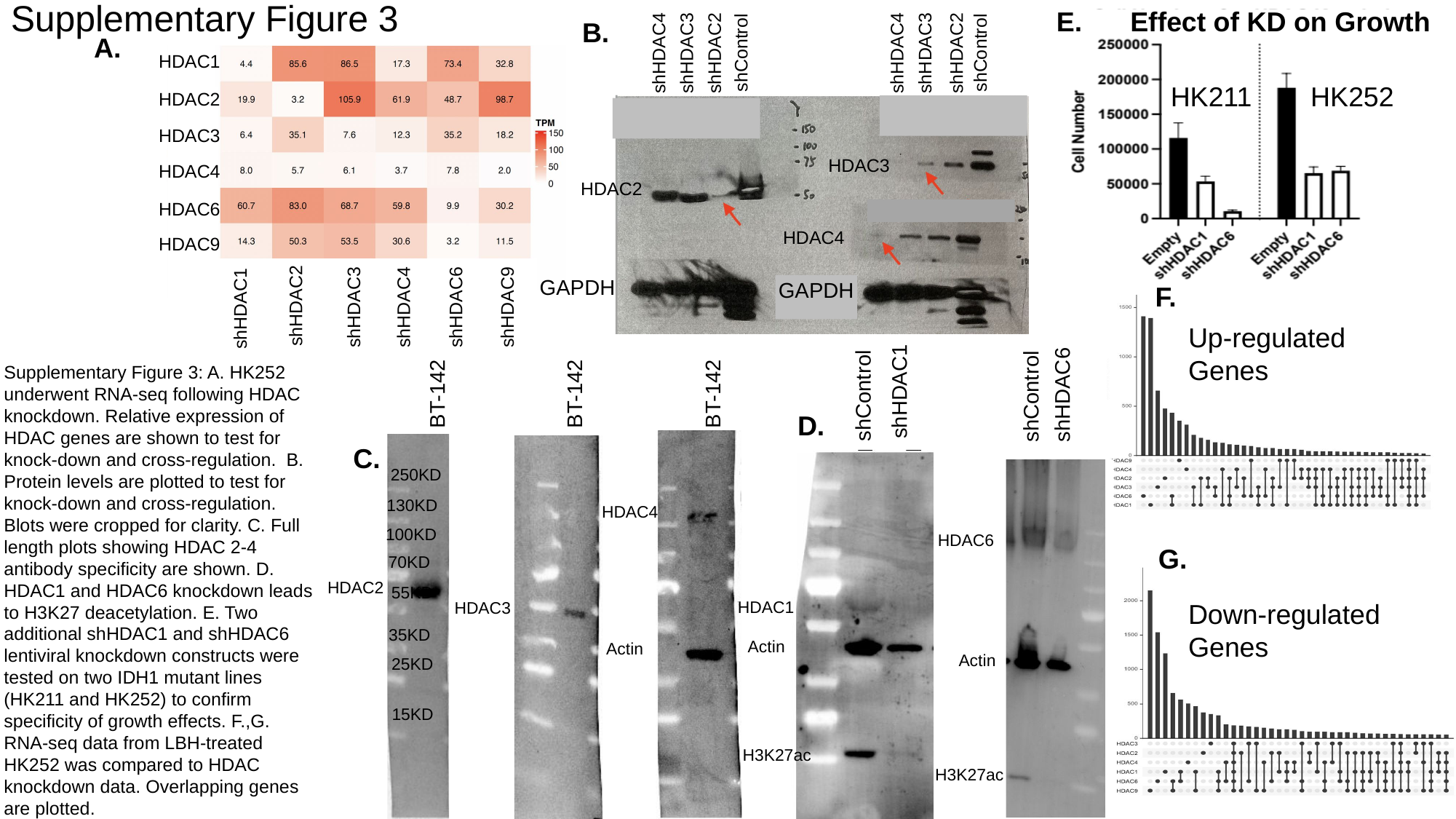

Supplementary Figure 3
Effect of KD on Growth
E.
B.
A.
shHDAC4
shHDAC3
shHDAC2
shControl
shHDAC4
shHDAC3
shHDAC2
shControl
HDAC1
HK211
HK252
HDAC2
HDAC3
HDAC3
HDAC4
HDAC2
HDAC6
HDAC4
HDAC9
GAPDH
GAPDH
F.
shHDAC2
shHDAC3
shHDAC4
shHDAC9
shHDAC6
shHDAC1
Up-regulated Genes
Supplementary Figure 3: A. HK252 underwent RNA-seq following HDAC knockdown. Relative expression of HDAC genes are shown to test for knock-down and cross-regulation. B. Protein levels are plotted to test for knock-down and cross-regulation. Blots were cropped for clarity. C. Full length plots showing HDAC 2-4 antibody specificity are shown. D. HDAC1 and HDAC6 knockdown leads to H3K27 deacetylation. E. Two additional shHDAC1 and shHDAC6 lentiviral knockdown constructs were tested on two IDH1 mutant lines (HK211 and HK252) to confirm specificity of growth effects. F.,G. RNA-seq data from LBH-treated HK252 was compared to HDAC knockdown data. Overlapping genes are plotted.
shHDAC1
BT-142
BT-142
BT-142
shHDAC6
shControl
shControl
D.
C.
250KD
130KD
HDAC4
100KD
HDAC6
G.
70KD
HDAC2
55KD
HDAC1
HDAC3
Down-regulated Genes
35KD
Actin
Actin
Actin
25KD
15KD
H3K27ac
H3K27ac
